# A new brilliantly blue-emitting luciferin-luciferase system from *Orfelia fultoni* and Keroplatinae (Diptera)

**DOI:** 10.1038/s41598-020-66286-1

**Published:** 2020-06-15

**Authors:** Vadim R. Viviani, Jaqueline R. Silva, Danilo T. Amaral, Vanessa R. Bevilaqua, Fabio C. Abdalla, Bruce R. Branchini, Carl H. Johnson

**Affiliations:** 1 Graduate School of Biotechnology and Environmental Monitoring (UFSCar), Federal University of São Carlos (UFSCar), Sorocaba, Brazil; 2 Graduate School of Evolutive Genetics and Molecular Biology (UFSCar), São Carlos, Brazil; 3 Department of Chemistry, Connecticut College, New London, Connecticut USA; 4 Dept. Biological Sciences, Vanderbilt University, Nashville, TN, USA

**Keywords:** Photobiology, Oxidoreductases

## Abstract

Larvae of *O. fultoni* (Keroplatidae: Keroplatinae), which occur along river banks in the Appalachian Mountains in Eastern United States, produce the bluest bioluminescence among insects from translucent areas associated to black bodies, which are  located mainly in the anterior and posterior parts of the body. Although closely related to *Arachnocampa spp* (Keroplatidae: Arachnocampininae), *O.fultoni* has a morphologically and biochemically distinct bioluminescent system which evolved independently, requiring a luciferase enzyme, a luciferin, a substrate binding fraction (SBF) that releases luciferin in the presence of mild reducing agents, molecular oxygen, and no additional cofactors. Similarly, the closely related *Neoceroplatus spp*, shares the same kind of luciferin-luciferase system of *Orfelia fultoni*. However, the molecular properties, identities and functions of luciferases, SBF and luciferin of *Orfelia fultoni* and other  luminescent members of the Keroplatinae subfamily still remain to be fully elucidated. Using *O. fultoni* as a source of luciferase, and the recently discovered non-luminescent cave worm *Neoditomiya* sp as the main source of luciferin and SBF, we isolated and initially characterized these compounds. The luciferase of *O. fultoni* is a stable enzyme active as an apparent trimer (220 kDa) composed of ~70 kDa monomers, with an optimum pH of 7.8. The SBF, which is found in the black bodies in *Orfelia fultoni* and in smaller dark granules in *Neoditomiya sp*, consists of a high molecular weight complex of luciferin and proteins, apparently associated to mitochondria. The luciferin, partially purified from hot extracts by a combination of anion exchange chromatography and TLC, is a very polar and weakly fluorescent compound, whereas its oxidized product displays blue fluorescence with an emission spectrum matching the bioluminescence spectrum (~460 nm), indicating that it is oxyluciferin. The widespread occurrence of luciferin and SBF in both luminescent and non-luminescent Keroplatinae larvae indicate an additional important biological function for the substrate, and therefore the name *keroplatin*.

## Introduction

Bioluminescence among insects is found in Collembola, Diptera and mainly in Coleoptera^1^. Beetles like fireflies, click beetles and railroadworms produce a wide range of colors from green to red using the same bioluminescent system involving similar luciferases homologous to CoA-ligases, a benzothiazolic luciferin and ATP^1^. The bioluminescent system of beetles has been extensively used for bioanalytical purposes during the last decades^2^.

In Diptera, bioluminescence is found exclusively in the mosquito larvae of the family Keroplatidae, which includes the subfamilies Arachnocampininae with luminescent species in the Oceanic genus *Arachnocampa spp*, and Keroplatinae with luminescent species in the Euroasiatic genus *Keroplatus* spp and the Nearctic *Orfelia fultoni*^3^. Despite pertaining to the same family, *Arachnocampa* and *Orfelia fultoni* display morphologically and biochemically distinct bioluminescence systems.

Larvae of *Arachnocampa* spp, which construct webs in the roof of caves and emit blue-green bioluminescence to lure prey^4^, produce bioluminescence by a whitish lantern located in the tip of the abdomen, consisting of modified terminal ends of Malpighian tubules^5^, and involves a luciferase, a luciferin and ATP^6^. Proteomic and transcriptional studies suggest that in *Arachnocampa* species the luciferases are enzymes of the CoA-ligase superfamily, distantly related to beetle luciferases^7,8^. Despite a report, which claimed that the luciferin of *Arachnocampa richardsae* is identical to firefly (beetle) luciferin^9^, cross-reactions between *Arachnocampa* (AR) and firefly luciferin and luciferases performed by other research groups were unsuccessful^3,6^. Indeed, the chemical structure of *Arachnocampa luminosa* luciferin was recently shown to be a derivative of xanthurenic acid and tyrosine, clearly distinct from firefly luciferin^10^.

**Figure 1:**
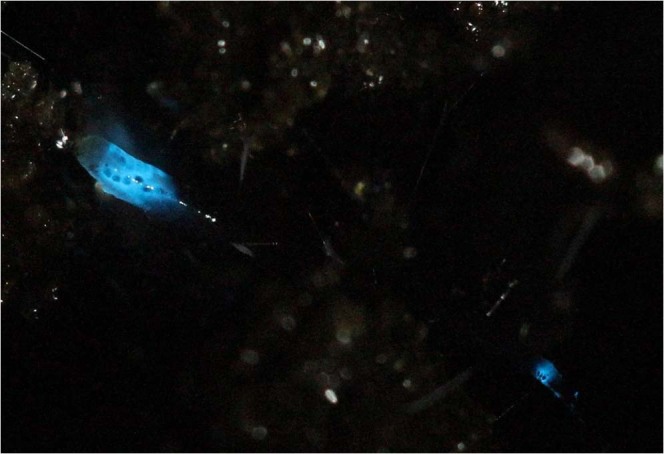
Larva of *Orfelia fultoni* bioluminescence. Note that bioluminescence surrounds the black bodies.

Larvae of *Orfelia fultoni*, which construct webs along moisty stream banks and falls along the Appalachian mountains in the Eastern United Stated (Fig. 1)^11^, emit the bluest bioluminescence (460 nm) among insects (Fig. 1)^3^ by translucent areas associated to rows of black bodies mainly from the anterior and posterior parts of the body,^12^ also with the goal to lure preys. The biochemical system was shown to involve a 140 kDa dimeric luciferase, a very unstable luciferin and a substrate binding fraction (SBF) which apparently releases luciferin in the presence of mild reductants such as DTT and ascorbate^3^. The absence of cross-reaction between *Arachnocampa* and *Orfelia* luciferins and luciferases, as well as morphological differences between their lanterns, leave little doubt that bioluminescence arose independently and evolved at least twice in the Keroplatidae family, once in Arachnocampininae and another time in Keroplatinae subfamilies^3^. Curiously, we recently found that the non-luminescent larvae of *Neoditomiya* sp, that lives inside Brazilian Atlantic rain forest caves, has *Orfelia*-type luciferin and the substrate binding fraction (SBF), but no luciferase, indicating that presence of *Orfelia*-type luciferin could be a common ancestral trait in the Keroplatinae subfamily and that bioluminescence may have evolved later^13^.

Finally, larvae of *Keroplatus* spp, live on dead logs and are apparently sporofagous instead of carnivorous, giving them the name of true fungus-gnats^14,15^. They display structures resembling *Orfelia* black bodies and bioluminescence in the blue region with spectral peak also close to that of *Orfelia* (~460 nm)^15^, but no biochemical study has ever been conducted. Very recently, the first South-American bioluminescent species was discovered in the Atlantic rain forest in Brazil, *Neoceroplatus betharyensis*^16^, which is closely related to *Keroplatus* spp. Luciferin-luciferase cross-reaction and molecular studies, conducted in our laboratory, showed that *Neoceroplatus* and *Orfelia fultoni* share the same bioluminescence system and that these species are closely related, providing evidence that bioluminescence in the Keroplatinae subfamily evolved a single time^16^.

The absence of cross-reaction of *Orfelia* luciferin-luciferase with any other known bioluminescent system, except those from the same subfamily, attest that the luciferase and luciferin are novel. However, the molecular properties and identities of luciferin, luciferase and SBF in Keroplatinae species remain unknown, in part, due to the lack of abundant and readily accessible sources of biological material. Therefore, in this study we first purified and characterized the molecular properties of the luciferase from *Orfelia fultoni* larvae collected in North Carolina, USA. Then, using the purified luciferase from *Orfelia fultoni,* and the non-luminescent *Neoditomiya* sp larvae as the main source of SBF and luciferin, we investigated the anatomical location and molecular constitution of SBF. Finally, we partially isolated and characterized the spectroscopic properties of the luciferin and its oxidation product. Combination of proteomic and transcriptional analysis also identified putative candidates for the luciferase and SBF.

## Results and Discussion

### Anatomical and cellular sources of luciferin and SBF

#### Orfelia fultoni

In *Orfelia fultoni*, bioluminescence is produced mainly by the anterior and posterior parts of the body, while a weaker level of bioluminescence^3^ is produced throughout the body. Bioluminescence was associated to rows of black bodies^12^, however, it is not yet clear whether these are true photocytes or if they are related to bioluminescence.

**Figure 2:**
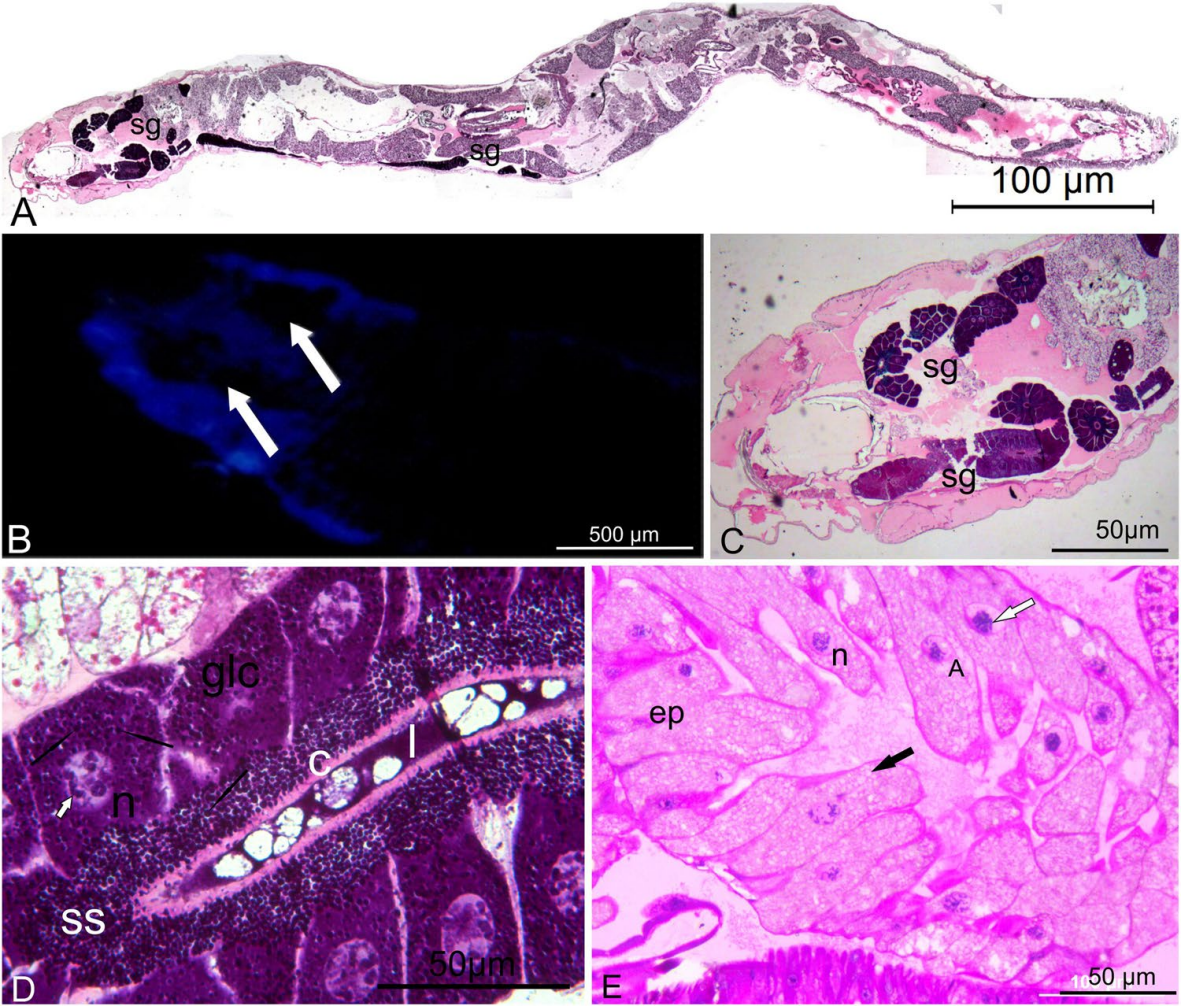
(**A**) Longitudinal section of the *Orfelia* larvae, showing paired tubular structures like the silk glands (sg) from the anterior to the posterior part of the larvae, HE. (**B**) Total preparation of the cephalic-thorax of *Orfelia* larvae under fluorescence. Notice blue fluorescence surround the black structures (white arrows). (**C**) Cephalic-thorax section showing that the black structures saw in B are the silk glands (sg), HE. (**D**) Detail of the glandular cells (glc) of *Orfelia* larvae filled with basophil granules (black arrows), which are released to the subcuticular space (ss), crossing the cuticle (c) into the lumen (l). Notice the polytenic chromosomes (white arrow) into the nucleus (n). (**E**) Histological preparation of the epithelial cells (ep) of *Neoditomiya* larvae, showing polytenic chromosomes inside the nuclei (n) and vacuolization like (black arrow) into cytoplasm.

The larvae of three specimens were studied showing symmetrical paired tubular structures located alongside the thoracic portion (Fig. 2), extending to the mid-posterior portion of the abdomen. These structures are dark pigmented and the surrounding areas emit a weak blue fluorescence, as shown by fluorescence microscopy (Fig. 2). The morphological analysis of these structures suggest that they could be silk glands, also called larval salivary glands in Diptera^17^. However, we did not observe any structure like photocytes as previously described by Bassot^12^. From the morphological analysis, we found that the blackish anterior structures were part of a very active glandular epithelium, which had cells filled with protein-derived basophilic intracellular granules (Fig. 2). The cells also showed mechanisms of gene amplification, such as polytene chromosomes (Fig. 2), which are typical of silk and salivary glands of other insects^17^. The glandular cells release the intracellular granules first into the subcuticular space through an apocrine like-mechanism, and then into the lumen, where they become a very basophil, homogenous like matrix (Fig. 2). According Azuma and Ohta^18^ in *Bombix morio* larvae, the glandular cells of the posterior portion of the silk gland have a proton-translocating vacuolar-type ATPase (V-ATPase). This biochemical system creates an intracellular compartment at the apical portion of the epithelial cells able to acidify the extracellular environment by pumping out protons of the cell. It is not surprising that in our proteomic analysis of partially purified luciferase we also found V-ATPase.

**Figure 3:**
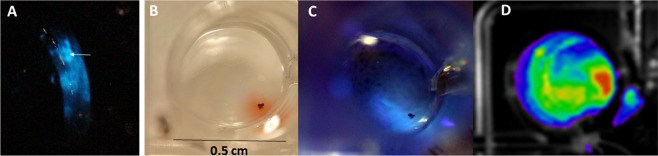
Bioluminescence, pigmentation and fluorescence associated with *Orfelia fultoni* black bodies: (**A**) *Orfelia* thorax evidencing bioluminescent areas surrounding the black bodies; (**B**) DTT-induced release of brownish-orange pigment; (**C**) associated blue fluorescence released by black bodies upon threatment with DTT and UV irradiation and (**D**) CCD-image of bioluminescence associated with the black bodies.

Clearly, while the areas surrounding the black bodies could be bioluminescent, the black bodies themselves cannot be. In fact, when we analyzed the photographic images of the photogenic area, we found that bioluminescence is produced only as rings surrounding such black bodies (Fig. 3).

To better understand the function of black bodies in *Orfelia fultoni* bioluminescence, we isolated these structures from live *Orfelia* larvae and analyzed their natural bioluminescence in saline solution and in the presence of DTT, luciferase, and DTT + luciferase (Fig. 3). Initially, the isolated black bodies display bioluminescence around them, either in the absence of DTT or luciferase, as a consequence of the presence of luciferase and luciferin. Addition of DTT, however, increases the bioluminescence. It is noteworthy that upon the addition of DTT in the saline solution, the black bodies begin to release a dense brownish-orange pigment, similar to that already observed for the hot extracts and for the pigment adsorbed to surfaces during luciferin isolation steps. The released pigment by the black bodies, also displays intense blue fluorescence upon irradiation with UV light (375 nm), which could be associated to oxyluciferin produced by luciferin oxidation in the presence of luciferase, as we will show later. Also, the addition of purified luciferase to the black body saline solution that had been treated with DTT for several minutes produced strong bioluminescence. These results indicate that the black bodies consist of larger aggregates of SBF, and constitute the source which stores and release the reduced luciferin for the bioluminescence reaction catalyzed by the surrounding luciferase.

#### *Neoditomiya* sp

In the case of the non-luminescent larval *Neoditomiya sp*, the source of luciferin is unclear because these larvae do not have the same large black bodies such as observed in *Orfelia fultoni* larvae. However, these larvae display two dark pigmented lines along the dorsal part of the body, which are constituted by dark pigmented cells. Notably, these cells, (Fig. 2E), resemble those of the dorsal portion of *Arachnocampa flava* light organ described by Rigby and Merritt^19^, which are constituted by specialized portions of Malpighian tubules

**Figure 4:**
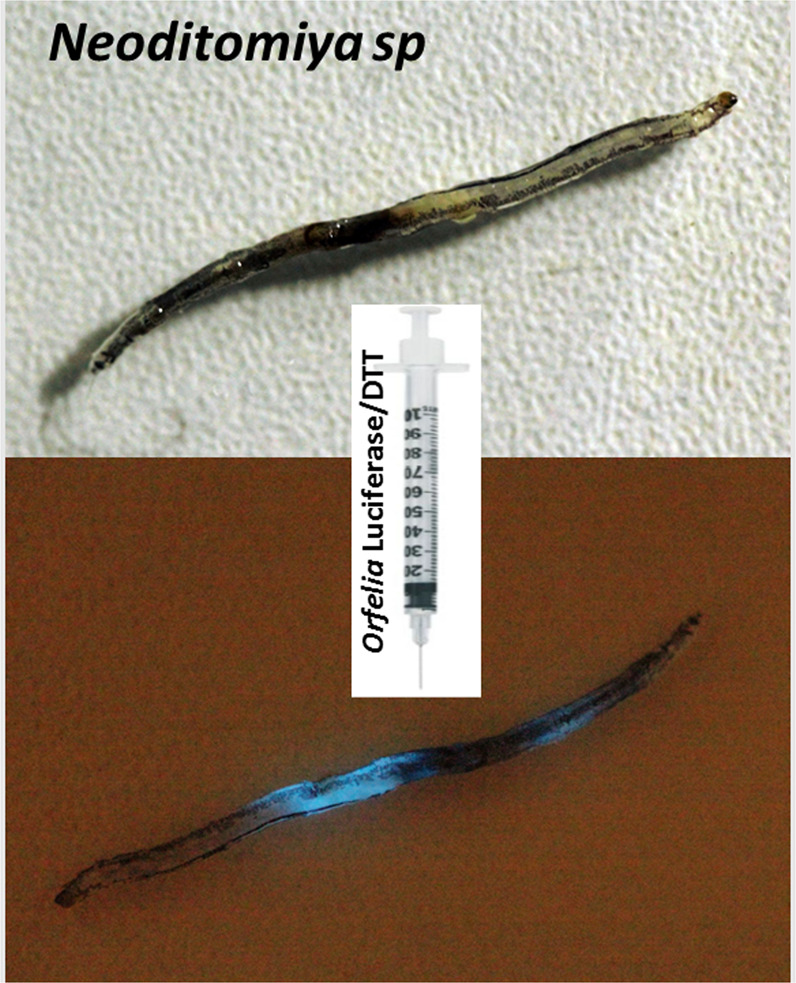
Induced bioluminescence in *Neoditomiya* larvae upon injection of *Orfelia* luciferase and DTT.

To determine the anatomical distribution of luciferin and SBF, we first injected a solution of purified *Orfelia* luciferase plus DTT inside the body of living larvae, and analyzed the bioluminescence by CCD imaging and photography. We found that intense visible bioluminescence is generated throughout the whole body (Fig. 4), indicating that SBF and luciferin are widespread along the body of the larva.

We then isolated the fat body, Malpighian tubules and the dark pigmented dorsal cells found in *Neoditomiya* body in isotonic solution, and threated them with a solution of purified *Orfelia* luciferase in the presence of DTT. CCD camera analysis revealed that only the dorsal dark granules generated bioluminescence, indicating that these granules are indeed the source of luciferin and SBF.

The only apparent common trait between *Orfelia* black bodies and *Neoditomiya* dark pigmented cells, is the presence of pigmentation, however, it remains unclear what is the structural and functional relationship between these structures. More studies are necessary to confirm the identity of such structures and their histological relationships.

#### Cellular location of SBF

**Figure 5:**
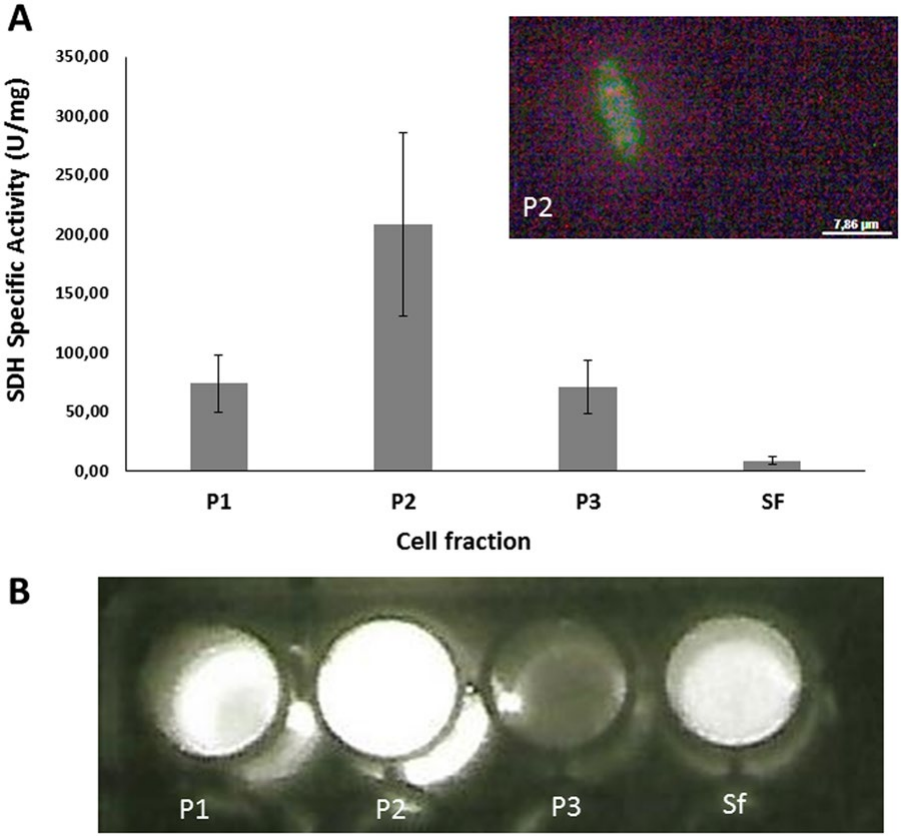
Cellular location of SBF in *Neoditomiya* sp larvae: (**A**) graph of succinate dehydrogenase (SDH) specific activity of cellular fractions. The upper right inset shows the intact mitochondria visualized by fluorescence microscopy; (**B**) bioluminescent activity of SBF upon mixing extracted cell fractions with *Orfelia* purified luciferase and 5 mM DTT; (P1) nuclear enriched fraction; (P2) mitochondrial enriched fraction; (P3) peroxisomal enriched fraction and (Sf) final supernatant.

In order to better define the intracellular location of SBF, and therefore luciferin in *Neoditomiya* larvae, we made cell fractionation and analyzed the presence of SBF as well as of succinate dehydrogenase (SDH) activity as a mitochondrial marker. Interestingly, SBF specific activity was higher in the mitochondrial enriched fraction (P2), as can be seen by its higher bioluminescence upon addition of DTT and luciferase (Fig. 5). The presence of mitochondria in P2 fraction was also verified by fluorescence microscopy. The results indicate the association of SBF with the mitochondria, supporting the previous ultrastructural analysis by Bassot^12^ with *Orfelia fultoni,* which associated the black bodies with mitochondrial origin. However, we cannot discard the possibility that SBF, being constituted by large molecular aggregates, may display a similar sedimentation coefficient as mitochondria.

### *Orfelia* Luciferase purification and properties

#### Purification and Initial Characterization

Previous studies showed that the active luciferase, isolated by molecular exclusion has molecular weight of 140 kDa, and SDS PAGE showed two main bands of about 70 kDa^3^. Here we further purified and investigated the molecular weight and other properties of *Orfelia* luciferase using distinct approaches.

We first used combined ammonium sulfate precipitation, molecular filtration and anion exchange chromatography, to obtain luciferase enriched-fractions. As expected, molecular filtration experiments using molecular 100 kDa *cut off* filters, showed that most of the luciferase activity remained in the retentate. However, the filtrate also displayed weak luciferase activity, about 10–15% of the retentate activity, indicating that the monomeric form(s) could also be active, or more likely, that some of the monomeric form(s) may re-associate to form the active enzyme.

**Figure 6:**
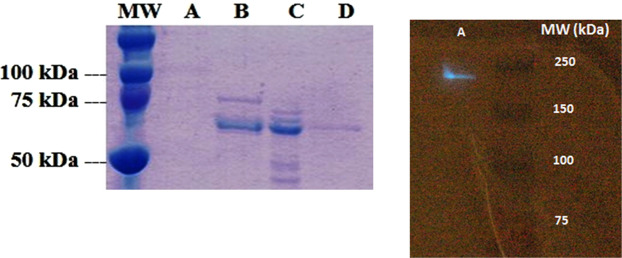
(Left) SDS-PAGE purification steps of the *Orfelia* luciferase: (**A**) 0–40% ammonium sulphate precipitated fraction; (**B**) 40–75% ammonium sulphate luciferase-enriched fraction; (**C**) 200–300 mM NaCl anion-exchange chromatography eluted luciferase and (**D**) luciferase isolated from Native-PAGE (right panel), concentrated and re-electrophoresed on denaturing SDS-PAGE; (Right): Photographic image of the Native-Page of *Orfelia* partially purified luciferase: (**A**) lane of purified *Orfelia* luciferase. The luminescence of the luciferase band was revealed after spreading *Orfelia* hot extract over the gel following by photography with a Canon Ei5 camera upon exposure during 20 s at high sensitivity (3200 ISO).

After ammonium sulfate precipitation, the 40–75% luciferase-enriched fractions were re-dissolved in 25 mM Tris-HCL pH 8.0 buffer, then dialyzed against the same buffer, and finally separated by spin column anion exchange chromatography with a 100–500 mM NaCl gradient. The luciferase eluted preferentially in the 200–300 mM NaCl fractions. The SDS-PAGE analysis of the purification steps performed here showed that both the 40–75% ammonium sulfate precipitated and the 200–300 mM NaCl eluted luciferase-enriched fractions, contained 5 bands with MW ranging between 70–80 kDa (Fig. 6). In both fractions, two lower molecular weight bands of ~70 and ~74 kDa were more intense than in the 0–40% ammonium sulfate and the 100 mM NaCl fractions, which are enriched by SBF, indicating that the ~70 and ~74 kDa MW bands may correspond to the luciferase.

To further investigate quaternary structure, we also analyzed the luciferase-enriched fraction isolated by anion-exchange chromatography by functional assay on Native-PAGE electrophoresis (Fig. 6). Notably, only a single band of ~220 kDa exhibited strong bioluminescence. We then isolated the bioluminescent 220 kDa gel bands from Native PAGE, eluted the protein from the gel and re-electrophoresed the protein on denaturing SDS-PAGE. Moreover, in the SDS-PAGE, a single ~70 kDa band appeared. Altogether, the above purification and electrophoresis approaches indicate that *Orfelia* luciferase consists of a trimer of ~70 kDa monomers.

**Figure 7:**
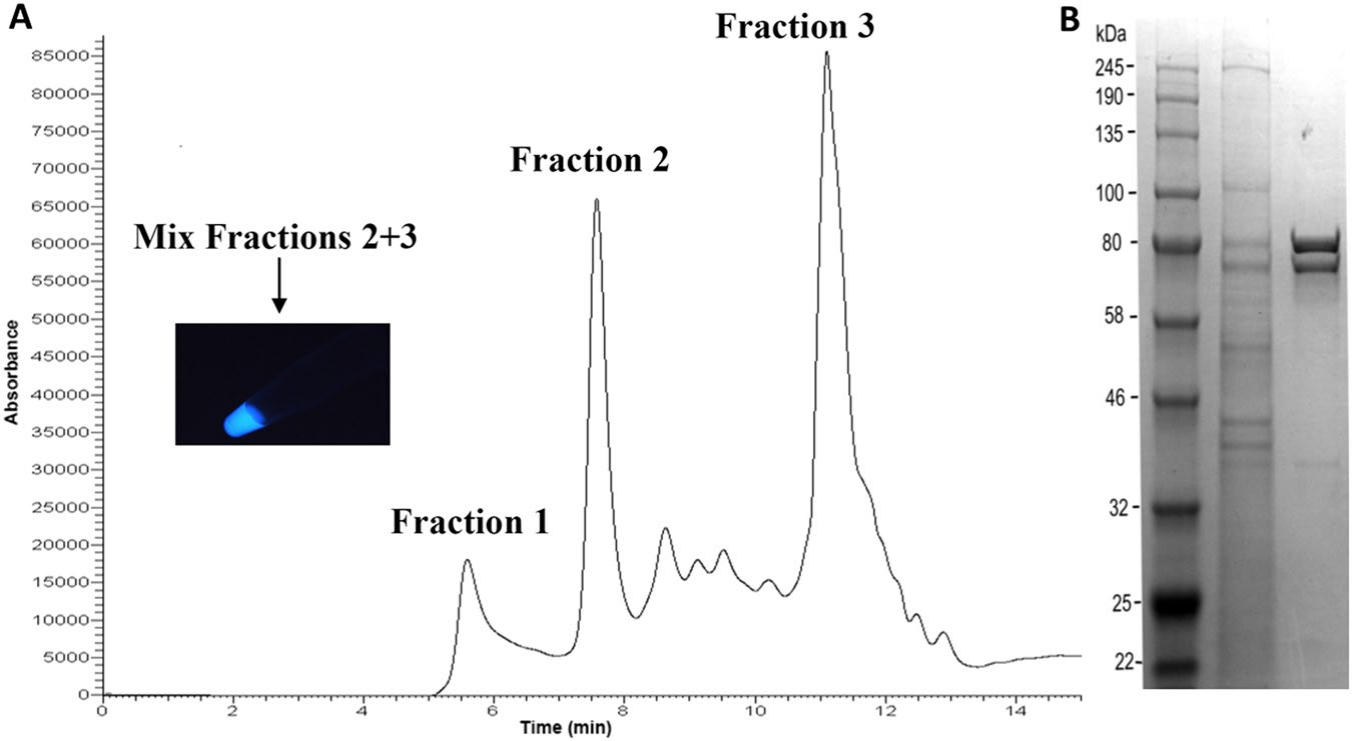
(**A**) Separation of an *Orfelia fultoni* larvae extract and bioluminescence of luciferin- and luciferase- containing fractions. An extract of 4 frozen *Orfelia* larvae was prepared by grinding the organisms in liquid N_2_, mixing with 0.10 M pH 7.0 ammonium acetate buffer containing 1 mM EDTA. After centrifugation, 75 uL of the supernatant was applied to a Yarra 300 × 4.6 mM SEC-3000 size exclusion column eluted at a flow rate of 1 mL/min on a Thermo Finnegan Surveyor HPLC system with total UV monitoring. The inset shows a photograph taken in the dark of 0.4 mL of Fractions 2 and 3 mixed together in an Eppendorf tube; (**B**) SDS-PAGE of an *Orfelia fultoni* larvae extract before and after size exclusion chromatography. The crude larvae extract (middle lane) and luciferase activity-containing concentrated Fraction 2 from the experiment illustrated in panel A were applied and run on a gradient SDS gel.

In a separate set of experiments, we analyzed *Orfelia fultoni* larval extracts using high pressure liquid chromatography (HPLC) with a Yarra 300 × 4.6 mm SEC-3000 size exclusion column (Fig. 7). We verified that the breakthrough (>700 kDa, Fraction 1) contained SBF based on the release of active luciferin with DTT and other reducing agents. Fraction 2 (~240 kDa) contained luciferase activity and a late eluting Fraction 3 (<10 kDa) contained luciferin activity. Surprisingly, mixing the enzyme and substrate containing fractions produced light without the addition of reducing agents. However, we later determined that Fraction 3 also contained GSH, likely explaining why additional reducing agents were not required, and how the readily oxidized substrate maintained functionality.

Further analysis of Fraction 2 by HPLC ion exchange chromatography produced a fraction with luciferase activity that upon SDS-PAGE analysis revealed intense bands of ~80 kDa, and ~74 kDa, as well as a minor band at ~37 kDa (Fig. 7).

#### Optimum pH, Stability, and Bioluminescence spectra

**Figure 8:**
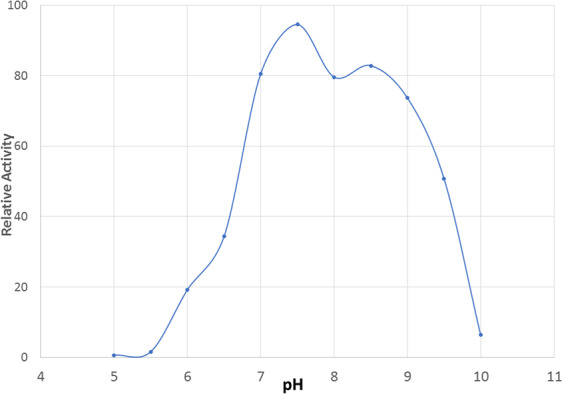
Effect of pH on luminescence activity of purified *Orfelia* luciferase.

The luciferase has strong activity over a broad pH range 7.0 to 8.5 with optimal activity at ~pH 7.8 (Fig. 8). Notably, the enzyme also shows high bioluminescent activity at low temperatures between 0–4 °C consistent with observed *in vivo* bioluminescent activity of *Orfelia* larvae in the field during the cold early spring nights. Furthermore, the partially purified enzyme was very stable, retaining ~80% activity between pH 7.0 and 9.0 (Fig. 8), and retaining full activity for more than 4 months when stored at 4 °C in Tris-HCl buffer at pH 8.0 (Table 1).

**Table 1:** *Orfelia* luciferase and SBF properties.

Protein	MW (kDa)	MW monomer (kDa)	Activity (10^6^ cps/mg)	pH	Stability (Days)*	λ_max_ (nm)
Luciferase	220	~70	4900	7.8	>120	463
SBF	>700	~65/75	—			—

*In 0.2 M NaCl 25 mM Tris-HCL pH 8.0.

**Figure 9:**
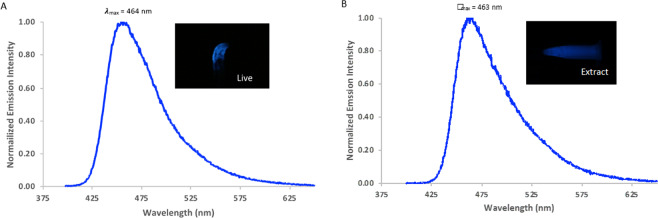
Bioluminescence spectra of *Orfelia* (**A**); *in vivo* and (**B**) *in vitro*.

The BL spectra measured with a liquid N_2_ cooled CCD imaging device and with a spectrofluorometer, confirmed that the emission of the live organism, cold extract of frozen organisms and partially purified luciferase were essentially identical displaying maxima of 463 ± 1 nm (Fig. 9), as previously shown^3^.

#### Proteomic characterization

In order to find out the molecular identity of the luciferase, we used proteomic analysis of SDS-PAGE and Native-PAGE isolated luciferase bands and cross-checked against the recently obtained transcriptional data-base of *Orfelia fultoni* larvae (Table S2).

The mass-spectrometry analysis of the SDS-PAGE (70 kDa) and Native-PAGE (220 kDa) isolated luciferase bands showed that the most common protein hits for luciferase were hexamerin-like proteins with considerable identity with arthropod hemocyanins. The most abundant protein hits found were hexamerins type 1 and 2. Other common hits, identified by MS analysis of the single protein bands, were Hsp70, tropomyosin, ATP-synthase type V (as previously shown in SEC isolated samples), Hsp90, Glucose dehydrogenase and NADPH cytP450. Although the molecular weight of hexamerins monomeric forms is usually above 80 kDa, which is higher than the expected molecular weight of luciferase bands, it shall be noted that these proteins often have a N-terminal signal sequence which is removed by proteolysis, resulting in nearly 70 kDa polypeptides.

On the other hand, the proteomics analysis of the excised bands from the SDS-PAGE gel of the SEC purified luciferase produced good matches for the ~74 kDa bands (22–26% coverage) to V-type proton ATPase catalytic A subunits from mosquitos *Aedes aegypti* and *Anopheles gambiae*. For the ~37 kDa band (28–50% coverage) matches to tropomyosins from a variety of sources including *Drosophila melanogaster* and *Bombyx mori* were found. Unfortunately, the ~80 kDa produced no matches to the NCBI data base.

The hemocyanins are two copper center containing proteins, usually associated with oxygen binding and storage in arthropods hemolymph^20^, with exception of insects. However, in a few cases hemocyanin was also reported in basal clades of insects^21^, suggesting that some insect basal taxa, and perhaps the larval stage of some clades, may also display hemocyanins or hemocyanin-like proteins. Hemocyanins are also basal members of a larger family of proteins which include insect hexamerins, which are oligomeric proteins forming larger aggregates (300–500 kDa), originally associated to nutritional storage proteins in insects, and later associated with a wider range of functions, including riboflavin binding, hormone binding and immune response^21^.

Besides binding oxygen, hemocyanins may also display alternative phenol- and diphenol-oxidase activities, which may characterize them as monooxygenases, a functional requirement for luciferases. However, we could not detect phenoloxidase activity *Orfelia* luciferase using typical substrates DOPA and syringaldizine (results not shown), in contrast with commercial mushroom tyrosinase and laccase positive controls.

While we can’t rule out the hexamerins/haemocyanins or V-ATPase subunit V as candidates for the luciferase, it is plausible that the enzyme could be a unique gene product with no match in the protein data bank.

#### Substrate binding fraction (SBF)

A third high molecular weight bioluminescence system component eluting near the void volume, the Substrate Binding Fraction (SBF), identifiable by its bioluminescence activation upon DTT (or other mild reducing agents such as ascorbate and GSH) addition in the presence of luciferase, was previously found^3^. Previous studies showed that *Orfelia* SBF has a MW > 500 kDa, results which are confirmed also here by SEC studies which showed a MW > 700 kDa.

However, the molecular nature of SBF and mechanism of action remained unknown. It was unclear whether SBF could be a specific luciferin binding protein or a reductase. The presence of luciferin binding proteins (LBP) already has been described in bioluminescent organisms such as dinoflagellates, in which the luciferin is a very readily oxidizable compound that must be stored and protected from autooxidation^22^. In dinoflagellates, a drop in pH modulates the release of luciferin.

**Figure 10:**
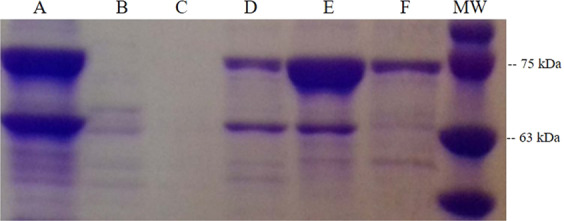
SDS-PAGE of the purification steps of *Neoditomiya* sp SBF: (**A**) crude extract; (**B**) outflow from anion exchange chromatography; (**C**) wash; (**D**) 100 mM NaCl; (**E**) 200 mM NaCl; (**F**) 300 mM NaCl and (MW) molecular weight markers. SBF activity predominates between the 100 and 200 mM NaCl eluted fractions.

We first attempted to isolate and characterize SBF using ammonium sulphate precipitation, and anion-exchange chromatography (Fig. 10) of the recently discovered non-luminescent cave-worm *Neoditomiya*, as a main biological source of luciferase-free SBF and *Orfelia*-type luciferin. During the 0–40% ammonium sulfate fractionation of *Neoditomiya* (or *Orfelia*) crude extracts, the SBF remained in a jelly-like supernatant. Subsequent molecular filtration using 100 kDa *cut off* filters further separated the components (Fig. 10).

SDS-PAGE analysis showed that both the 40% ammonium sulfate fraction and the 100 mM NaCl eluted ion exchange fraction contain high molecular weight protein bands (~66–75 kDa; Fig. 10).

Mass-spectrometry analysis of the SDS-PAGE isolated SBF protein bands from both *Orfelia* and *Neoditomiya* also showed that the most common protein hits were hexamerin-like proteins. The presence of hexamerins is consistent with the formation of high molecular weight aggregates by SBF (MW > 500 kDa) and the molecular weight of SBF monomeric protein bands found in SDS-PAGE analysis (66 and 75 kDa). But it is unclear whether hexamerins could play a specific role in luciferin binding or reduction.

**Figure 11:**
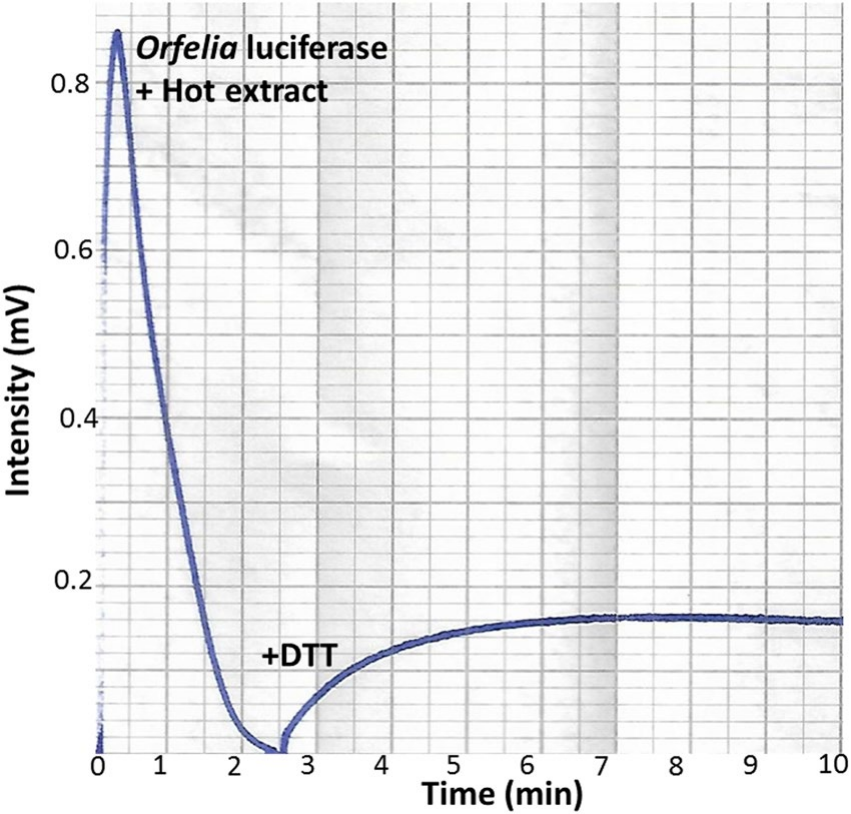
Kinetics of bioluminescent reaction upon mixing partially purified *Orfelia fultoni* luciferase and hot extract (without DTT), and later addition of DTT. Addition of DTT brings about an gradual increase of bioluminescence to the almost spent reaction.

To determine whether a specific protein of SBF could be involved in luciferin binding or release, or as a reductase, we prepared protein-free larval hot extracts in the absence of DTT at acidic conditions (to avoid luciferin oxidation in the absence of DTT). As expected, mixing purified *Orfelia* luciferase with these extracts promoted bioluminescence, with flash-like luminescence kinetics (Fig. 11) faster than the kinetics observed upon mixing luciferase with the hot extracts prepared in the presence of DTT. Surprisingly, the later addition of DTT to the spent bioluminescence reaction, produced a slow build up of bioluminescence reaching high intensity (Fig. 11), indicating that DTT has a direct reducing effect on the luciferin in the hot extracts, independently of the presence of proteins. If the hot extract was incubated in Tris-HCl buffer pH 8.0, in the presence of DTT during 5 min at room temperature, and then mixed with the luciferase, a higher BL intensity and more sustained luminescence was observed, confirming that DTT has a direct reducing effect on the hot extract luciferin, and not on the luciferase.

Altogether, the results shown here indicate that SBF is not a specific luciferin binding protein, nor a reductase, but rather a high molecular weight complex of oxidized luciferin associated with oligomeric proteins and other cellular components, mainly mitochondria.

Noteworthy, in the Australasian *Arachnocampa* spp larvae, which display a distinct bioluminescent system from *Orfelia fultoni*, the lanterns also display a high content of hexamerins^7,8^. Therefore, whereas the presence of hexamerins/haemocyanin-like proteins in either the luciferase and SBF fractions of *Orfelia* and *Neoditomiya* sp could be just the result of the widespread abundance of hexamerins in the larval stage of insects, it quite likely that hexamerin-like proteins may play an important role in the bioluminescence systems of Keroplatidae.

### Luciferin isolation and properties

#### General properties of hot extracts and luciferin

We measured the stability of luciferin in the hot extracts at different pHs. The hot extracted luciferin was very unstable above pH 7.0, being more stable at pH<6.0. DTT was shown to be essential to keep the luciferin reduced and more stable in solution. We could keep the luciferin quite stable for several months in blood collecting-type vessels under light vacuum at −20 °C.

The hot extracts display an intense brownish-orange color at pH 7, and more purplish at more alkaline pH, indicating presence of pigments. SDS-PAGE analysis confirmed that the hot extracts are protein-free (results not shown), and therefore only low molecular weight compounds including pigments are present. After air oxidation the hot extracts become more yellowish.

As expected for most luciferins, the active *Orfelia* luciferin displays a MW lower than 3 kDa, as indicated by filtration using 3 kDa MW filters, and by the SEC studies. However, as previously published and as shown here by SEC, luciferin can also be found as a high molecular weight complex, that we originally called SBF.

The luciferin can be extracted from larvae with DMSO or methanol in the presence of DTT, however, DMSO is more efficient. The luciferin could not be extracted from acidified aqueous hot extracts by acidic ethyl acetate, indicating that the luciferin is very polar and water-soluble molecule.

#### Separation and purification of Orfelia and Neoditomiya luciferins

**Figure 12:**
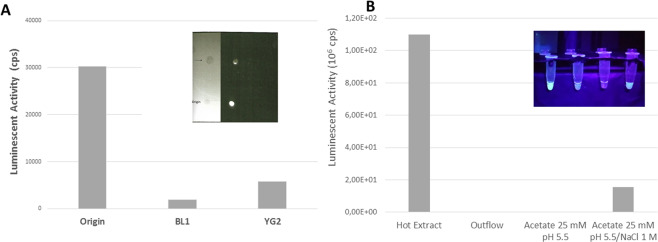
Separation of *Orfelia* luciferin**: (A)** Chemiluminescent TLC of *Orfelia* hot extract using the solvent system Ethyl Acetate/Ethanol/Water (5:3:2) and respective luminescent activity of TLC bands eluted in H_2_O/5 mM DTT upon addition of purified luciferase. (upper inset) The bioluminescence on TLC was revealed after adding purified luciferase over the migration path, and imaged using CCD-camera system. (BL1) blue-fluorescent spot with lower migration coefficient eluted from TLC, and (YG2) yellow-green fluorescent spot with higher migration coefficient and bioluminescent activity eluted from TLC. Note that most of bioluminescence activity is retained mainly on the origin of the migration path; (**B**) bioluminescence activity of anion exchange chromatography fractions; (upper inset) fluorescence of anion exchange eluted fractions (from left to right): Hot extract; outflow; wash in 25 mM acetate pH 5.5; elution with acetate 25 mM pH 5.5/ 1 M NaCl/DTT 5 mM.

We could partially separate and isolate luciferin from *Orfelia* and *Neoditomiya* hot extracts by anion exchange spin column chromatography using a strong anion exchanger and by TLC (Fig. 12).

During anion-exchange chromatography using centrifugation Q-Minicolumns, the orange-brownish pigmentation remained stuck on the surface of the membrane. The flow-through volume of the first centrifugation step displayed a green-yellow fluorescent compound (λ_FL_ = 543 nm). Partial elution of the luciferin was accomplished only with an acidic 1 M NaCl solution/DTT (Fig. 12). However, only about 10% of the luciferin recovered in the active form, whereas most of the active luciferin remained stuck on the membrane together with the orange-brown pigment. These results indicate that *Orfelia* luciferin has strong affinity for polar surfaces indicating a very polar and possible acidic nature.

We also standardized a chemiluminescent TLC technique using bioluminescence to detect luciferin on TLC plates, which consisted of applying purified *Orfelia* luciferase over the migration path of luciferin on TLC plates, and revealing the bioluminescence using a sensitive CCD-camera (Fig. 12). Upon applying the hot extract on the silica-gel, a mixed blue/yellow-green fluorescent spot could be observed under UV light irradiation (375 nm) at the origin. After chromatography, however, the fluorescence split into two migrated spots, one with a higher Rf value and a more green-yellowish fluorescence and another one with a smaller Rf value and bluish fluorescence, whereas the origin completely lost the fluorescence.

Under most conditions, using different solvent systems [ethyl acetate/etanol/water (5:3:2); ethyl acetate/etanol/water (3:5:2); methanol 70%] and silica gel plates, most of the luciferin of both *Orfelia* and *Neoditomiya* stuck at the origin, as can be seen from the much brighter bioluminescent spot at the origin of silica-gel, whereas a much lower amount migrated with the high Rf fluorescent spots (Fig. 12), as could be confirmed by the weaker bioluminescence upon applying luciferase. Notably, similar results were obtained by reverse-phase TLC using C18-coated silica-gel plates.

It is noteworthy that during the isolation procedures using either silica-gel-TLC or anion-exchange spin column chromatography, most of the luciferin bioluminescence activity was always retained together with the brownish-orange pigment on the surfaces of stationary phases. These results indicate that the brownish-orange pigment may correspond to the native form of the luciferin, and confirms that *Orfelia* luciferin is a very polar molecule with strong interaction with polar surfaces. This strong interaction to surfaces may also provide a plausible explanation for the association of luciferin to high molecular weight complexes with proteins in the SBF.

#### UV and Fluorescence spectra of Orfelia-type luciferin

The fresh hot extracts of *Orfelia* display bluish fluorescence, whereas those of *Neoditomiya* display a somehow more yellow-green fluorescence under UV light. *Orfelia* hot extracts incubated at RT and at 4 °C in presence of air, naturally display more intense blue fluorescence, whereas *Neoditomiya* hot extracts exposed to air during several days display more intense blue-green fluorescence.

During ammonium sulphate fractionation and other luciferase purification procedures, the blue fluorescence was always recovered in the supernatants, indicating that the blue-fluorescent compound must be very polar and also water soluble.

After scraping and eluting in water/DTT 5 mM the adsorbed compounds retained in the TLC origin, which contains most of the active luciferin, and in the migrated fluorescent spots on the TLC silica-gel plates, we measured the UV and fluorescence spectra.

**Figure 13:**
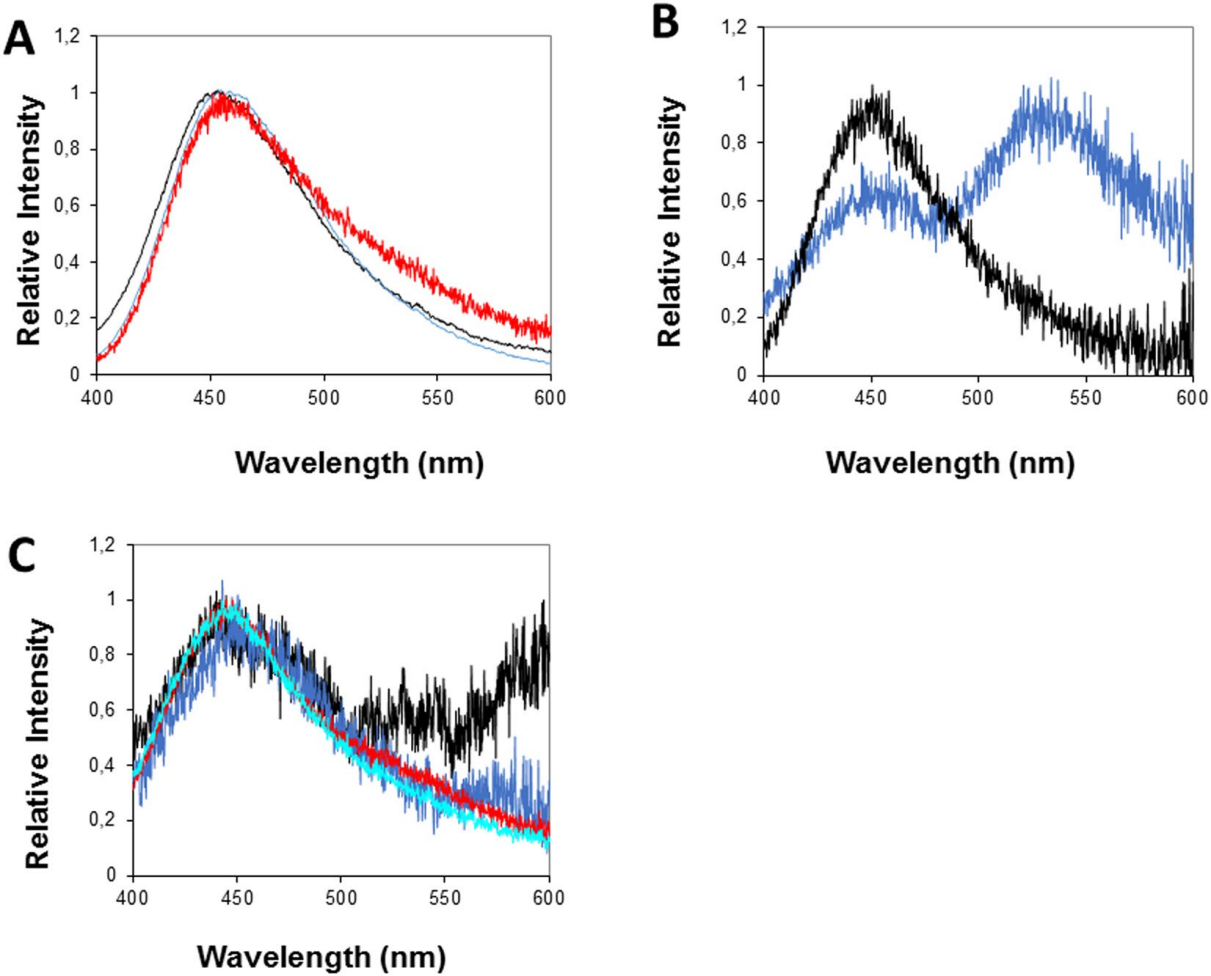
Normalized fluorescence spectra of *Orfelia* and *Neoditomiya* hot extracts and isolated luciferins: (**A**) hot extracts: (black) *Orfelia fultoni*; (Blue) *O. fultoni* air oxidized hot extract and (Red) *Neoditomiya* hot extract; (**B**) Anion exchange isolated luciferin fractions from *Neoditomiya* hot extracts: (Black) 1 M eluted luciferin and (Blue) wash out; (**C**) TLC isolated samples from *Neoditomiya* sp hot extract: (Black) eluted luciferin retained at the origin; (Dark Blue) eluted luciferin retained at the origin after air oxidation; (Red) eluted fluorescent spot and (Light blue) eluted fluorescent spot after air oxidation.

As expected, the blue fluorescent (BL1) migrated spot eluted fraction displayed more intense blue fluorescence (λ_EX_ = 360 nm; λ_FL_ = 457 nm), whereas the non-migrated eluted material retained at the origin, which contains most of the luciferin, was much less fluorescent and displayed a slightly more blue-shifted emission spectrum (λ_FL_ = 443 nm; Table 2). The luciferin eluted from the origin displayed an excitation spectrum with a main peak at 360 nm and a minor one at 290 nm, and emission spectrum with peak at ~457 nm (Fig. 13; Table 2), and UV spectrum with absorption peaks of 257 and 347 nm. The blue-fluorescent eluted spot showed a main absorption peak at 235 nm and a second peak around 340 nm.

**Table 2:** Chromatographic and spectroscopic properties of *Orfelia* and *Neoditomiya* hot extracts and partially isolated luciferins.

Fraction	Rf* solv. I	Rf solv. II	λ_abs_ (nm)	λ_EX_ (nm)	λ_FL_ (nm)**
*Orfelia* hot extract	—	—		362	457
*Neoditomiya* hot extract	—	—		362	458
Hot extract air oxidized	—	—		362	459
TLC-eluted origin	0	0	257/347	360	442
TLC-eluted origin (oxidized)			251/350	360	460
TLC-eluted band 1	0.66	0.74	—	360	443
TLC-eluted band 2	0.77	0.8	—	360	447/~540
Qcolumn-Eluted Luciferin	—	—	254/365	356	447
Oxidized Qcolumn-Eluted luciferin	—	—		362	457

*These values represent the average of 3 independent measurements.

**The reported values are average of at least 3 independent experiments, and the error associated with peak estimation is ±2.5 nm.

Similarly, the anion exchange of hot extracts, revealed two fluorescent components: one with a fluorescence spectrum peak in the green-yellow region (λ_FL_ = 543 nm) and a minor second peak in the blue region (λ_FL_ ~450 nm) which came out in the flow-through of the first centrifugation step (Fig. 12B), and another corresponding to the eluted luciferin with 1 M NaCl/DTT which displayed weak bluish fluorescence (λ_FL_ ~450 nm; Fig. 12). The UV spectrum of such anion exchange eluted sample displayed a main peak at 254 nm and a second one at ~365 nm.

Spontaneous air- and luciferase-catalyzed oxidation of either the *Orfelia* or *Neoditomiya* hot extracts at room temperature showed that the blue fluorescence gradually increases over time and becomes slightly more red-shifted (λ_FL_ = 457–460 nm). Similarly, the eluted material from the origin of the TLC or from anion-exchange had almost no fluorescence in the beginning, but after air exposure for 1–2 hours at room temperature gradually acquired more intense fluorescence in the blue region. The fluorescence spectra in such cases matched the fluorescence spectrum of *Orfelia* hot extracts and of the TLC isolated fluorescent spots (457 nm), indicating that the blue fluorescence arises from an oxidized form of the luciferin. Because the fluorescence spectrum of the oxidized form matches quite well the bioluminescence spectrum of *Orfelia* luciferin-luciferase system (λ_BL_ = 463 nm), the results give compelling evidences that the blue-fluorescent compound corresponds to the oxyluciferin, the emitter of the bioluminescent reaction. Such results are also consistent with the more intense blue fluorescence observed for both the crude and hot extracts of *Orfelia*, which naturally contain luciferase which oxidizes luciferin, in contrast to *Neoditomiya* crude extracts that do not contain active luciferase and display a more yellow-green fluorescence.

#### Keroplatin: a new luciferin with other biological functions

As previously suggested^16^, the widespread occurrence of *Orfelia*-type luciferin in either luminescent and non-luminescent larvae of Keroplatinae subfamily, clearly indicate that this compound may display alternative biochemical roles in these organisms. This is not the first case of a luciferin which is found in both luminescent and non-luminescent organisms, since coelenterazine luciferin is widespread in the marine organisms, but surely it is the first case in the terrestrial organisms. So far, firefly luciferin has been found only in luminescent beetles. Since *Orfelia*-type luciferin is not limited only to bioluminescent larvae, we propose the use of the more generic name *keroplatin*, instead of luciferin. It is not clear yet what is the possible biological function of keroplatin in non-luminescent larvae, but because it is associated to black bodies or pigmented cells, it is possible that it could be a pigment associated to the process of melanization in these larvae, or perhaps an antioxidant molecule. The participation of other known luciferins such as coelenterazine as antioxidants, was already proposed^23^. Alternatively, because the black bodies are associated to salivary glands, which are involved in silk production, we could not exclude the possibility that keroplatin could be an essential co-factor in such process.

## Concluding remarks

The luciferase of *Orfelia fultoni*, and the luciferin and SBF from *Orfelia* and the non-luminescent *Neoditomiya* sp were partially purified and characterized. The luciferase is a stable oligomeric enzyme, with an optimum pH near 7.8, which is active mainly as a 220 kDa trimer composed by ~70 kDa monomers. SBF is a high molecular weight complex of luciferin associated with proteins and mitochondria, which participate in the constitution of the black bodies in *Orfelia fultoni* and dark pigmented cells in non-luminescent *Neoditomiya*. The black bodies are therefore the source of rduced luciferin for the bioluminescent reaction. The reduced luciferin is a polar, unstable (readily oxidized) and weakly fluorescent compound, whereas the oxidized form it is very polar and water soluble, displaying intense blue fluorescence matching the bioluminescence spectrum. The widespread occurrence of luciferin, in both luminescent and non-luminescent larvae of Keroplatinae subfamily, indicates additional biological functions for this compound, and therefore we propose the use of the more general name *keroplatin*. Studies are ongoing to identify the chemical structures of such luciferin and the putative oxyluciferin, and to further elucidate its biological functions.

## Methods

### Insects

*Orfelia fultoni* larvae were collected in May of 2015, 2016, 2017 and 2018 along stream and river banks near the Biological Station of Highlands and Dry Falls at the National Forest of Nantahala, Highlands-NC (Fig. 1). The larvae were collected, frozen and stored in a −80 °C cooler and brought to our laboratories at the Vanderbilt University (Nashville-TN) and Federal University of São Carlos, Brazil. Larvae of *Neoditomiya* sp were collected in the roof of the caves at Intervales Park near Guapiara (São Paulo state, Brazil) in April-December 2017–2019.

### CCD imaging

Bioluminescence imaging of larvae, pupae and tissues and isolated structures was done using a LightCapture II (ATTO, Tokyo) CCD camera system at high sensitivity, or a NightOwl II CCD camera system (Berthold, Germany).

### Cell microscopy

For histological analyses, larvae of *Arachnocampa* sp., *Neoditomiya* sp. and *Orfelia fultoni* were fixed in 4% paraformaldehyde for 24 h, dehydrated with a graded ethanol series (15%, 25%, 50%, 70%, 80%, 85% and 95%, 20 min. each). After, the material was diaphanized xilol:ethanol PA (1:1) 20 min., and xilol PA (24 h). Upon diaphanization, the larvae were embedded (Leica Biosystems Nussloch GmbH, Heidelberg, Germany), according to the manufacturer’s recommendations. Histological sections of 2 µm thickness were cut with a Leica microtome (RM 2255) and stained with hematoxylin and eosin (Merck). The material was analyzed using a Leica light microscope (DM 1000). Total preparation of *Orfelia fultoni* larvae was also observed under a Leica Fluorescence photomicroscope (DM4000) with B/G/R fluorescence optical system, ultraviolet (BP 465/20), blue (BP 530/30) and green (BP 640/40) excitation bands.

### Cell fractionation

Cell fractionation was performed according to Lemos and Terra^24^. In brief, 4 *Neoditomyia* larvae were homogenized in cold isotonic solution using a Potter Elvejem. The homogenate was centrifuged once at 750 g during 5 min at 4 °C. The pellet was resuspended in 1 mL of cold isotonic solution and centrifuged again to obtain the pellet P1 (enriched nuclear fraction). The supernatants were mixed and centrifuged at 3,600 g during 10 min to obtain the pellet P2 (enriched mitochondrial fraction). The resulting supernatant was then centrifuged at 20,000 g during 20 min to obtain the peroxisomal fraction. All pellets were resuspended in 500 µl of cold extraction buffer, sonicated with 2 pulses using an ultrasonicator, and centrifuged at 15,000 g during 15 min. The resulting supernatants were used to assay SBF luciferin release and succinate dehydrogenase (SDH) activity as a mitochondrial enzyme marker.

### SDH assay

Succinate dehydrogenase (SDH) activity was spectrophotometrically assayed according an adaptation of Lemos and Terra *et al.*^24^ of the original method of Ackrell *et al.*^25^.

### Luciferase activity

Luciferin and luciferase reactions were assayed according *Orfelia*,s luciferin-luciferase assays^3^. Luminescence intensity was measured by integration during 10 s in *cps* (counts per second) using an AB2200 luminometer (ATTO, Tokyo). For luciferase activity, 5 µl of hot extract containing luciferin were mixed with 90 µl of 0.10 M Tris-HCL buffer pH 8.0 and 5 µl of *Orfelia* luciferase in the luminometer tube.

### SBF activity

The SBF activity was assayed by mixing 5–10 µl of SBF preparation to 85–90 µl of 0.10 M Tris-HCl buffer pH 8.0 and 5 µl of *Orfelia* purified luciferase, and finally adding 5 µl of 100 mM DTT. The activity was measured in the luminometer as the increase of luminescence activity in counts per second (cps) upon adding DTT.

### Total protein concentration

Estimation of total protein concentration of the dipteran crude extracts was made using Bradford methodology, after obtaining a standard curve with  BSA.

### Luciferase and SBF purification

We succeeded in purifying the luciferase using a limited amount of available biological material, by using a combination of ammonium sulfate precipitation, molecular filtration and ion exchange chromatography. We used 4–5 larvae/mL of extraction buffer for each purification. The larvae were extracted in sodium phosphate buffer pH 7.0 supplemented with 1 mM EDTA, 1% Triton X-100 and centrifuged at 15,000 g during 15 min at 4 °C. The supernatant containing the luciferase and SBF was then treated with ammonium sulfate up to 40% saturation, and centrifuged. The resulting pellet was saved and the supernatant was treated with ammonium sulfate up to 70% saturation, and centrifuged again. The luciferase containing precipitate was then resuspended in 25 mM Tris-HCl buffer pH 8.0, and dialyzed overnight against 1 L of the same buffer. The dialyzed fraction was then used for anion exchange chromatography using Mini or Maxi Q spin columns (Sartorius). The fractions eluted between 200–300 mM NaCl contained most of the active luciferase (Fig. 4). For larger scale purification, the luciferase was eluted in a single step using 300 mM NaCl. SDS-PAGE of the purification steps are shown in (Fig. 4). SBF purification followed the same scheme described above using *Orfelia* larvae, which contains both luciferase and SBF. However, to avoid interference of *Orfelia* luciferase, which often co-elutes with SBF, we used non-luminescent *Neoditomiya* larvae which are free from luciferase.

### SEC

We also used high pressure liquid chromatography (HPLC) with a Yarra 300 ×4.6 mm SEC-3000 column. Separation of an *Orfelia fultoni* larvae extract and bioluminescence of luciferin- and luciferase- containing fractions. An extract of 4 frozen *Orfelia* larvae was prepared by grinding the organisms in liquid N_2_, mixing with 0.10 mM pH 7.0 ammonium acetate buffer containing 1 mM EDTA. After centrifugation, 75 uL of the supernatant was applied to a Yarra 300 × 4.6 mM SEC-3000 size exclusion column eluted at a flow rate of 1 mL/min on a Thermo Finnegan Surveyor HPLC system with total UV monitoring.

### Native-PAGE

Native PAGE gels of *Orfelia* luciferase were run using 6% polyacrilamide gels according to Bio-Rad protocol. The luciferase samples were mixed with sample buffer without SDS and mercaptoethanol, and then applied on the gel, and electrophoresed on an ice-cooled apparatus at 150 volts during 1.5 hr using 25 mM Tris/192 mM glycine running buffer. Luciferase activity in the gel was revealed by bioluminescence upon spreading *Orfelia* hot extract over the gel during 5 min, and exposing it to a CCD camera, or by photographic analysis.

### Bioluminescence spectra

Bioluminescence spectra were recorded using a LumiSpectra spectroluminometer (ATTO, Tokyo), a Hitachi F4500 spectrofluorometer, and with a home made CCD-imaging device. During the assay in the spectroluminometer, 5–10 µl of luciferase preparation and 5 µl of hot extract were mixed to 90 µl of 0.10 M Tris-HCl buffer pH 8.0. For the spectrofluorometer, the above volumes were increased 5 times to a final volume of 500 µl.

### Proteomic analysis

In order to find out the molecular identity of the luciferase and SBF, the partially purified enzyme and SBF were electrophoresed in 7.5% SDS-PAGE, and the resulting protein gel bands isolated for proteomic analysis (Fig. S1; Table S1). After electrophoresis, the SDS gels were stained with colloidal blue, taking care to avoid keratin contamination. The amount of protein of each band was estimated to be between 50–500 ng. These bands were carefully excised from the gel, briefly washed with 50% acetonitrile, and stored in microfuge tubes in dry ice. The samples were shipped to Prof. D. Hayes McDonald from the Proteomics laboratory at Vanderbilt University for MS analysis. Proteomic analysis of the isolated gel samples and identification of known protein matches was performed using Scaffold 4 software^26^ and BLASTp.

### Transcriptional analysis

Total RNA was extracted using Trizol reagent (Life Technologies, USA) from the whole body of six large *Orfelia* larvae; three for each tested condition (triplicates), during the night (high bioluminescent activity period) after the collection, and the others during the day (low bioluminescent activity period). The RNA extraction quality was checked by spectrophotometry using a *NanoDrop* spectrophotometer (Thermo Scientific, USA) and *Agilent 2100 Bioanalyzer* (Agilent Tech., USA). The mRNA isolation and the cDNA library constructions were performed at VANTAGE facility (Vanderbilt University, TN, USA), using *TruSeq RNA Sample Preparation Kit* (Illumina Inc., USA). The six tagged cDNA libraries were pooled in equal ratios and used for 2 × 150 bp paired-end sequencing on a single lane of the Illumina HiSeq3000, according to the manufacturer’s instructions.

The reads were checked by FastQC 0.11.5 software^27^ and modified using FASTX-TOOLKIT 0.0.13. The cleaned reads were *de novo* assembled using Trinity 2.2.0^28^, in the default settings. The transcripts were subjected to similarity search agains t NCBI’s non-redundant (nr) and UNIPROT/SWISS-PROT databases in BLAST algorithm. The gene ontology (GO) terms and the pathways annotations using the Kyoto Encyclopedia of Genes and Genomes (KEGG) were determined by Blast2GO software^29^. We compared the abundance of genes/isoforms in each condition (night/day) using the FPKM values and we performed the differential expression (DE) analysis also comparing the normalized transcript abundance using the DESeq2 package^30^.

## Luciferin Isolation

### Hot extract preparation

The hot extract was prepared according to previously published *Orfelia* procedure^3,13^, by homogenizing 3 to 4 *Orfelia fultoni* or *Neoditomiya* sp larvae in Potter Elevejem containing cold ultrapure water or 0.10 M phosphate buffer pH 7.0, supplemented with 10 mM DTT. The extract was transferred into a glass tube sealed with a rubber cover, submitted to vacuum, and incubated at 98 °C during 5 min. After that, the hot homogenate was cooled in ice and centrifuged during 15 min at 15.000 g at 4 °C, and the supernatant, called *hot extract*, was stored under vacuum in a blood collecting-type vessel at −20 °C. Under such condition the luciferin could be kept stable for several months.

### TLC analysis

*Orfelia* and *Neoditomiya* luciferins were separated by TLC from hot extracts. We used both silica-gel and C_18_-coated silica gel plates, and a combination of solvent systems to elute the luciferin: (Solv. I) EtAc/EtOH/H_2_O (3:3:4); (Solv.II) EtAc/EtOH/H_2_O (2:5:3); (Solv.III) EtAc/EtOH/Sodium Acetate 25 mM pH 5.5/NaCL 1 M (2:3:5); (Solv. IV) Metanol/NaCl 1 M (7:3) and DTT 5 mM (7:3). We implemented a chemiluminescent TLC technique, in which luciferin bioluminescence could be revealed by CCD imaging after pipetting purified *Orfelia* luciferase solution onto the TLC migration path.

### Anion exchange chromatography

The luciferin from *Orfelia* and *Neoditomiya* hot extracts was partially purified by anion exchange chromatography. We tested Sartorius Mini Q, S, D, and C spin columns. The anion-exchange column Q showed to be the best choice.

### Fluorescence spectra

Fluorescence spectra of hot extracts, semi-purified luciferin from TLC plates or anion-exchange were obtained using a Hitachi F4500 spectrofluorometer. The excitation was scanned at RT from 250–400 nm using an excitation slit of 2.5 nm and emission slit of 10 nm, whereas the emission spectrum was scanned from 400–700 nm using the same values of excitation and emission slits. The spectra were autocorrected for the spectral response of the equipment.

## Supplementary information

Supplementary information.

## Data Availability

The RNA-seq raw reads of six samples have been submitted in BioSample Archive under project number PRJNA578979.
