# Author Correction: A new brilliantly blue-emitting luciferin-luciferase system from *Orfelia fultoni* and Keroplatinae (Diptera)

**DOI:** 10.1038/s41598-023-31188-5

**Published:** 2023-03-21

**Authors:** Vadim R. Viviani, Jaqueline R. Silva, Danilo T. Amaral, Vanessa R. Bevilaqua, Fabio C. Abdalla, Bruce R. Branchini, Carl H. Johnson

**Affiliations:** 1 Graduate School of Biotechnology and Environmental Monitoring (UFSCar), Federal University of São Carlos (UFSCar), Sorocaba, Brazil; 2 Graduate School of Evolutive Genetics and Molecular Biology (UFSCar), São Carlos, Brazil; 3 Department of Chemistry, Connecticut College, New London, CT USA; 4 Dept. Biological Sciences, Vanderbilt University, Nashville, TN USA

Correction to: *Scientific Reports* 10.1038/s41598-020-66286-1, published online 15 June 2020

The Data Availability section in the original version of this Article was omitted.

It now appears as below:

“The RNA-seq raw reads of six samples have been submitted in BioSample Archive under project number PRJNA578979.”

The original Article has been corrected.

