# Supplementary material for: A new brilliantly blue-emitting luciferin-luciferase system from Orfelia fultoni and Keroplatinae (Diptera)
Source: Sci Rep. 2020 Jun 15;10:9608. doi: 10.1038/s41598-020-66286-1 (PMC7295969; doi:10.1038/s41598-020-66286-1)
Supplement: Supplementary file 1 — Supplementary information. [file 41598_2020_66286_MOESM1_ESM.docx]

**A new brilliantly blue-emitting luciferin-luciferase system from *Orfelia fultoni* and Keroplatinae (Diptera)**

Vadim R. Viviani^1,2*^, Jaqueline R. Silva^1^, Danilo T. Amaral^1^, Vanessa R. Bevilaqua^2^, Fabio C. Abdalla^1^, Bruce R. Branchini^3^, Carl H. Johnson^4^

**
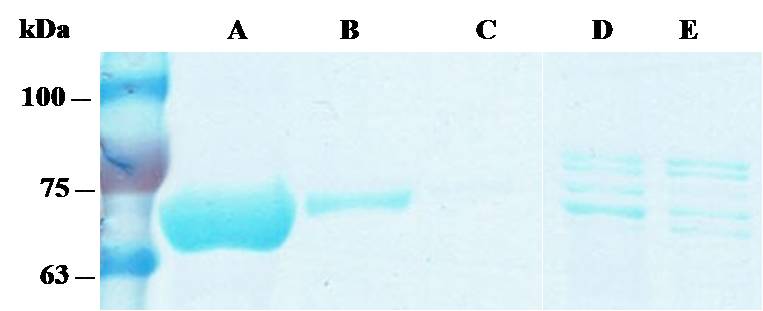
**

**Figure S1**. SDS-PAGE of purified *Orfelia fultoni* luciferase stained with colloidal-blue: (A) 5 µg BSA ; (B) 500 ng BSA; (C) 50 ng BSA; (D) purified luciferase (2/23/2016), (E) purified luciferase S300 (02/18/2016).

**Table S1**. Estimation of protein amount and molecular weight of SDS-PAGE isolated bands used for proteomic analysis (Supplemental material).

| **Sample** | **LANE D** |  | **LANE E** |  |
| --- | --- | --- | --- | --- |
|  | **Amount (ng)** | **MW (KDa)** | **Amount (ng)** | **MW (KDa)** |
| Band 1 | 418.42 | 81.3 | 348 | 81.3 |
| Band 2 | 476.78 | 77.6 | 342 | 77.7 |
| Band 3 | 443.0 | 74.1 | 298 | 74.1 |
| Band 4 | 501.75 | 72.4 | 327 | 72.4 |
| Band 5 |  |  | 309 | 66 |

**Table S2.** Protein hits identified from mass spectrometry analysis of *Orfelia fultoni* luciferase isolated from Native-PAGE gel bands.

| **Product** | **MW (kDa)** | **Protein** |
| --- | --- | --- |
| 1 | 89 | Hexamerin 2β |
| 2 | 85 | Hexamerin 1-1 like |
| 3 | 72 | Chaperone Hsp70 |
| 4 | 72 | Hsp70 |
| 5 | 224 | Myosin Heavy chain |
| 6 | 29 | Hexamerin |
| 7 | 46 | Hexamerin |
| 8 | 42 | Actin |
| 9 | 110 | Ca Transp. ATPase |
| 10 | 109 | Apoliphorin |
| 11 | 50 | Hexamerin |
| 12 | 624 | Twitchin |
| 68 | 68 | moesin |
| 14 | 40 | Arginine kynase |
| 15 | 82 | Hsp 82 |
| 16 | 55 | ATP-synthase |
| 17 | 68 | V ATP synthase |
| 18 | 70 | Glucose Dehyd. |
| 19 | 81 | peptidase |
| 20 | 82 | Threonine tRNA ligase |
| 21 | 75 | Hsp70 |
| 22 | 29 | Acidic Rib. protein |
| 23 | 52 | Succinate Dehydrogenase |
| 24 | 50 | α-tubulin |
| 25 | 36 | Malate Dehydrogenase |
| 26 | 91 | endoplasmin |
| 27 |  |  |
| 28 | 60 | Pyruvate Carb. MT isoform |
| 29 | 37 | serine-pyruvate aminotransferase |
| 30 | 104 | α-actin |
| 31 | 74 | Hexamerin |
| 32 |  |  |
| 33 |  |  |
| 34 | 99 | Filamin A |
| 35 | 94 | Elongation Factor 2 |
| 36 | 45 | Troponin T |
| 37 | 49 | Heat shock protein |
| 38 |  |  |
| 39 | 65 | Bifunct. Purine Bioss. Protein |
| 40 | 120 | Apoliphorin |
| 41 | 47 | Dissulfide isomerase |
| 42 | 50 | Elongation Factor |
| 43 | 50 | tubulin β |
| 44 | 60 | ATP-synthase |
